# Bis(2-dimethylamino-1,10-phenanthroline-κ^2^ *N*,*N*′)bis­(thio­cyanato-κ*N*)nickel(II) methanol disolvate

**DOI:** 10.1107/S1600536808011811

**Published:** 2008-05-03

**Authors:** Shi Guo Zhang, Tai Qiu Hu, Hong Li

**Affiliations:** a Department of Chemistry and Chemical Engineering, Institute of Materials Chemistry, Binzhou University, Binzhou 256603, People’s Republic of China; b Department of Chemistry, Shandong Normal University, Jinan 250014, People’s Republic of China

## Abstract

In the title complex, [Ni(NCS)_2_(C_14_H_13_N_3_)_2_]·2CH_3_OH, the Ni^II^ atom lies on a crystallographic twofold rotation axis and is in a slightly distorted octa­hedral NiN_6_ coordination environment. The crystal structure is stabilized by a combination of weak π–π stacking inter­actions between symmetry-related 1,10-phenanthroline ligands [centroi–centroid distance between benzene rings = 3.5936 (18) Å] and weak O—H⋯S, C—H⋯O and C—H⋯S hydrogen bonds between methanol and complex mol­ecules.

## Related literature

For related literature, see: Zhang *et al.* (2006 ▶); Liu *et al.* (2008 ▶).
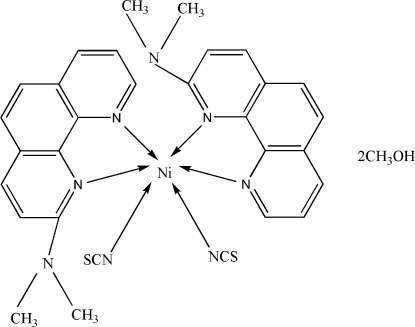


## Experimental

### 

#### Crystal data

[Ni(NCS)_2_(C_14_H_13_N_3_)_2_]·2CH_4_O
*M* *_r_* = 685.50
Monoclinic, 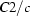

*a* = 19.573 (3) Å
*b* = 11.452 (3) Å
*c* = 16.338 (3) Å
β = 117.693 (4)°
*V* = 3242.6 (10) Å^3^
*Z* = 4
Mo *K*α radiation
μ = 0.77 mm^−1^
*T* = 298 (2) K
0.31 × 0.24 × 0.21 mm

#### Data collection

Bruker SMART APEX CCD diffractometer
Absorption correction: multi-scan (*SADABS*; Sheldrick, 1996 ▶) *T*_min_ = 0.796, *T*_max_ = 0.855
8459 measured reflections
3064 independent reflections
2668 reflections with *I* > 2σ(*I*)
*R*_int_ = 0.030

#### Refinement

*R*[*F* ^2^ > 2σ(*F* ^2^)] = 0.041
*wR*(*F* ^2^) = 0.100
*S* = 1.05
3064 reflections
210 parameters
H-atom parameters constrained
Δρ_max_ = 0.52 e Å^−3^
Δρ_min_ = −0.27 e Å^−3^

### 

Data collection: *SMART* (Bruker, 1997 ▶); cell refinement: *SAINT* (Bruker, 1997 ▶); data reduction: *SAINT*; program(s) used to solve structure: *SHELXTL* (Sheldrick, 2008 ▶); program(s) used to refine structure: *SHELXTL*; molecular graphics: *SHELXTL*; software used to prepare material for publication: *SHELXTL*.

## Supplementary Material

Crystal structure: contains datablocks I, global. DOI: 10.1107/S1600536808011811/lh2613sup1.cif

Structure factors: contains datablocks I. DOI: 10.1107/S1600536808011811/lh2613Isup2.hkl

Additional supplementary materials:  crystallographic information; 3D view; checkCIF report

## Figures and Tables

**Table:** 

N1—Ni1	2.0569 (19)
N2—Ni1	2.2556 (18)
N3—Ni1	2.047 (2)

**Table:** 

N3^i^—Ni1—N3	90.27 (11)
N3—Ni1—N1^i^	93.08 (7)
N3—Ni1—N1	88.63 (7)
N1^i^—Ni1—N1	177.57 (10)
N3—Ni1—N2	96.75 (7)
N1—Ni1—N2	77.31 (7)
N3—Ni1—N2^i^	167.90 (7)
N1—Ni1—N2^i^	100.76 (7)
N2—Ni1—N2^i^	78.13 (9)

Symmetry code: (i) 
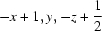
.

**Table 2:** Hydrogen-bond geometry (Å, °)

*D*—H⋯*A*	*D*—H	H⋯*A*	*D*⋯*A*	*D*—H⋯*A*
C14—H14*C*⋯S1^ii^	0.96	2.86	3.784 (3)	163
O1—H4⋯S1^iii^	0.82	2.65	3.331 (2)	142
C15—H15*B*⋯O1^iv^	0.96	2.51	3.427 (4)	161

Symmetry codes: (ii) 
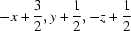
; (iii) 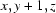; (iv) 
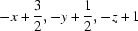
.

**Fig. 1.**
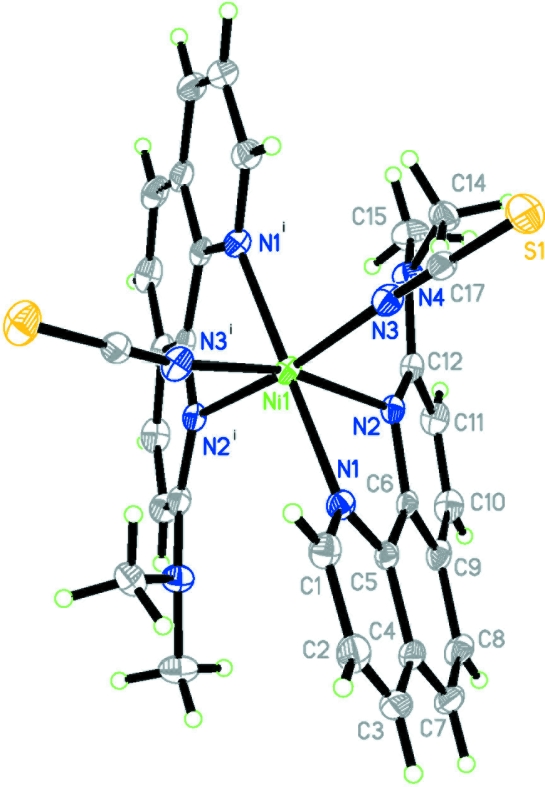
View of the molecular structure of complex (I), showing the the atom numbering scheme with thermal ellipsoids drawn at the 30% probability level (methanol molecules are not shown). Primed atoms are related by the symmetry operator (-x+1, y, -z+1/2).

**Fig. 2.**
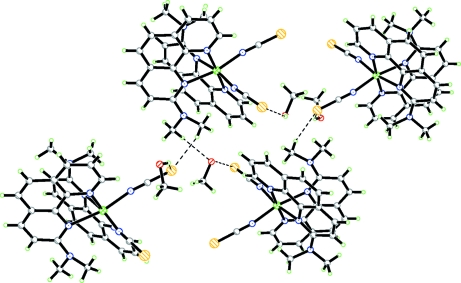
Part of the crystal structure showing hydrogen bonds between methanol molecules and complex molecules (dashed lines).

## supplementary crystallographic information

### Comment

The derivatives of 1,10-phenanthroline play a pivotal role in the area of modern coordination chemistry (Zhang *et al.* 2006) and a number of complexes have been reported with derivatives as ligands (Liu *et al.* 2008). Here we report the crystal structure of the title complex, (I), formed using 2-(dimethyl)amine-1,10-phenanthroline as a ligand.

The molecular structure of (I) is shown in Fig. 1. In the mononuclear complex, atom Ni1 is in a slightly distorted octahedral geometry (Table 1). There is a single π-π stacking interaction involving symmetry related 1,10-phenanthroline ligands, with the the relevant distances being *Cg*1···*Cg*1^i^ = 3.5936 (18) Å and *Cg*1···Cg1^i^_perp_ = 3.449 Å; α = 0.00° [symmetry code: (i) 1-*x*,-y,-*z*; *Cg*1 is the centroid of the C4—C9 ring; *Cg*1···*Cg*1_perp_ is the perpendicular distance from ring *Cg*1 to ring *Cg*1^i^; α is the dihedral between the two ring planes]. In addition, the crystal structure contains weak O—H···S, C—H···O and C—H···S hydrogen bonds between methanol molecules and complex molecules [Fig. 2 and Table 2]. In addition to the π-π stacking interactions and the hydrogen bonds there is relatively close contact between the H atom of the hydroxyl and symmetry-related pyridine ring [H···*Cg*2 = 2.82, where *Cg*2 is the centroid of N1/C1—C5 ring]. The combination of the above interactions help stabilize the crystal structure.

### Experimental

15 ml me thanol solution of Ni(ClO_4_).6H_2_O (0.2503 g, 0.684 mmol) was added into a 10 ml me thanol solution containing 2-(dimethl)amine-1,10-phenanthroline (0.1531 g, 0.686 mmol), and the mixed solution was stirred for a few minutes. Then 10 ml me thanol solution of NaSCN (0.1112 g, 1.37 mmol) was added into the mixed solution above. The green single crystals were obtained after the solution had been allowed to stand at room temperature for two weeks.

### Refinement

H atom of hydroxyl was located in a difference Fourier map and refined as riding in its as found position with  *U*_iso_(H) = 1.5 *U*_eq_(O). Other H atoms were placed in calculated positions (C—H = 0.96 Å for methyl group and C—H = 0.93 Å for other H atoms) and refined as riding with *U*_iso_ = 1.5 *U*_eq_(C) for methyl H and *U*_iso_ = 1.2 *U*_eq_(C) for other H atoms.

### Crystal data

**Table:**

[Ni(NCS)_2_(C_14_H_13_N_3_)_2_]·2CH_4_O	*F*_000_ = 1432
*M**_r_* = 685.50	*D*_x_ = 1.404 Mg m^−^^3^
Monoclinic, *C*2/*c*	Mo *K*α radiation λ = 0.71073 Å
Hall symbol: -C 2yc	Cell parameters from 3100 reflections
*a* = 19.573 (3) Å	θ = 2.4–27.8º
*b* = 11.452 (3) Å	µ = 0.77 mm^−^^1^
*c* = 16.338 (3) Å	*T* = 298 (2) K
β = 117.693 (4)º	Block, green
*V* = 3242.6 (10) Å^3^	0.31 × 0.24 × 0.21 mm
*Z* = 4	

### Data collection

**Table:**

Bruker SMART APEX CCD diffractometer	3064 independent reflections
Radiation source: fine-focus sealed tube	2668 reflections with *I* > 2σ(*I*)
Monochromator: graphite	*R*_int_ = 0.030
*T* = 298(2) K	θ_max_ = 25.7º
φ and ω scans	θ_min_ = 2.1º
Absorption correction: multi-scan(SADABS; Sheldrick, 1996)	*h* = −18→23
*T*_min_ = 0.796, *T*_max_ = 0.855	*k* = −12→13
8459 measured reflections	*l* = −19→19

### Refinement

**Table:**

Refinement on *F*^2^	Secondary atom site location: difference Fourier map
Least-squares matrix: full	Hydrogen site location: inferred from neighbouring sites
*R*[*F*^2^ > 2σ(*F*^2^)] = 0.041	H-atom parameters constrained
*wR*(*F*^2^) = 0.100	*w* = 1/[σ^2^(*F*_o_^2^) + (0.0522*P*)^2^ + 2.7575*P*] where *P* = (*F*_o_^2^ + 2*F*_c_^2^)/3
*S* = 1.05	(Δ/σ)_max_ < 0.001
3064 reflections	Δρ_max_ = 0.52 e Å^−^^3^
210 parameters	Δρ_min_ = −0.27 e Å^−^^3^
Primary atom site location: structure-invariant direct methods	Extinction correction: none

### Special details

**Table:**

Geometry. All e.s.d.'s (except the e.s.d. in the dihedral angle between two l.s. planes) are estimated using the full covariance matrix. The cell e.s.d.'s are taken into account individually in the estimation of e.s.d.'s in distances, angles and torsion angles; correlations between e.s.d.'s in cell parameters are only used when they are defined by crystal symmetry. An approximate (isotropic) treatment of cell e.s.d.'s is used for estimating e.s.d.'s involving l.s. planes.
Refinement. Refinement of *F*^2^ against ALL reflections. The weighted *R*-factor *wR* and goodness of fit *S* are based on *F*^2^, conventional *R*-factors *R* are based on *F*, with *F* set to zero for negative *F*^2^. The threshold expression of *F*^2^ > σ(*F*^2^) is used only for calculating *R*-factors(gt) *etc*. and is not relevant to the choice of reflections for refinement. *R*-factors based on *F*^2^ are statistically about twice as large as those based on *F*, and *R*- factors based on ALL data will be even larger.

### Fractional atomic coordinates and isotropic or equivalent isotropic displacement parameters (Å^2^)

**Table:**

	*x*	*y*	*z*	*U*_iso_*/*U*_eq_	
C1	0.38579 (14)	−0.2002 (2)	0.05726 (16)	0.0324 (6)	
H1	0.3846	−0.2706	0.0850	0.039*	
C2	0.33596 (15)	−0.1849 (2)	−0.03666 (17)	0.0385 (6)	
H2	0.3033	−0.2451	−0.0711	0.046*	
C3	0.33558 (15)	−0.0809 (3)	−0.07755 (17)	0.0371 (6)	
H3	0.3013	−0.0688	−0.1396	0.045*	
C4	0.38688 (14)	0.0075 (2)	−0.02593 (16)	0.0310 (5)	
C5	0.43824 (13)	−0.01749 (19)	0.06749 (15)	0.0248 (5)	
C6	0.49639 (13)	0.06546 (19)	0.12309 (15)	0.0234 (5)	
C7	0.38961 (16)	0.1199 (2)	−0.06162 (17)	0.0358 (6)	
H7	0.3562	0.1372	−0.1231	0.043*	
C8	0.43990 (15)	0.2013 (2)	−0.00747 (17)	0.0359 (6)	
H8	0.4391	0.2752	−0.0314	0.043*	
C9	0.49437 (14)	0.1764 (2)	0.08600 (16)	0.0296 (5)	
C10	0.54844 (15)	0.2582 (2)	0.14486 (18)	0.0351 (6)	
H10	0.5475	0.3344	0.1248	0.042*	
C11	0.60188 (15)	0.2262 (2)	0.23065 (17)	0.0336 (6)	
H11	0.6366	0.2811	0.2701	0.040*	
C12	0.60511 (14)	0.10882 (19)	0.26076 (16)	0.0269 (5)	
C14	0.69963 (14)	−0.0403 (2)	0.34968 (18)	0.0340 (6)	
H14A	0.6624	−0.0934	0.3064	0.051*	
H14B	0.7179	−0.0700	0.4113	0.051*	
H14C	0.7422	−0.0324	0.3362	0.051*	
C15	0.71573 (17)	0.1587 (2)	0.40742 (18)	0.0445 (7)	
H15A	0.7498	0.1885	0.3851	0.067*	
H15B	0.7455	0.1225	0.4666	0.067*	
H15C	0.6860	0.2216	0.4135	0.067*	
C16	0.5917 (2)	0.4773 (3)	0.3971 (3)	0.0682 (10)	
H6	0.5532	0.5318	0.3577	0.096 (14)*	
H9	0.5911	0.4728	0.4554	0.14 (2)*	
H5	0.5809	0.4016	0.3685	0.129 (19)*	
C17	0.59639 (13)	−0.3261 (2)	0.21965 (15)	0.0258 (5)	
N1	0.43477 (11)	−0.11804 (16)	0.10854 (12)	0.0257 (4)	
N2	0.55031 (11)	0.03107 (15)	0.20930 (12)	0.0231 (4)	
N3	0.56731 (12)	−0.24797 (17)	0.23466 (13)	0.0290 (4)	
N4	0.66394 (12)	0.07266 (17)	0.34233 (13)	0.0305 (5)	
Ni1	0.5000	−0.12186 (3)	0.2500	0.02153 (14)	
O1	0.66464 (13)	0.5147 (2)	0.41138 (16)	0.0681 (7)	
H4	0.6688	0.4940	0.3658	0.102*	
S1	0.63832 (4)	−0.43760 (6)	0.19758 (5)	0.0419 (2)	

### Atomic displacement parameters (Å^2^)

**Table:**

	*U*^11^	*U*^22^	*U*^33^	*U*^12^	*U*^13^	*U*^23^
C1	0.0360 (14)	0.0320 (13)	0.0322 (12)	−0.0045 (11)	0.0184 (11)	−0.0052 (10)
C2	0.0362 (15)	0.0467 (15)	0.0341 (13)	−0.0091 (12)	0.0177 (12)	−0.0138 (12)
C3	0.0300 (14)	0.0549 (16)	0.0236 (12)	0.0040 (12)	0.0102 (11)	−0.0049 (11)
C4	0.0321 (14)	0.0406 (14)	0.0246 (12)	0.0092 (11)	0.0167 (11)	0.0005 (10)
C5	0.0283 (12)	0.0275 (12)	0.0239 (11)	0.0060 (10)	0.0166 (10)	0.0002 (9)
C6	0.0283 (12)	0.0245 (11)	0.0247 (11)	0.0056 (9)	0.0186 (10)	0.0020 (9)
C7	0.0413 (15)	0.0440 (15)	0.0242 (12)	0.0139 (12)	0.0170 (11)	0.0085 (11)
C8	0.0494 (17)	0.0318 (13)	0.0339 (13)	0.0151 (12)	0.0256 (13)	0.0133 (11)
C9	0.0392 (14)	0.0246 (12)	0.0341 (12)	0.0067 (10)	0.0247 (12)	0.0048 (10)
C10	0.0466 (16)	0.0217 (12)	0.0459 (15)	0.0021 (11)	0.0291 (13)	0.0050 (11)
C11	0.0412 (15)	0.0265 (13)	0.0379 (14)	−0.0066 (11)	0.0225 (12)	−0.0033 (10)
C12	0.0316 (13)	0.0272 (12)	0.0286 (12)	−0.0013 (10)	0.0195 (10)	−0.0027 (9)
C14	0.0272 (13)	0.0349 (13)	0.0392 (14)	0.0001 (10)	0.0148 (11)	0.0060 (11)
C15	0.0419 (16)	0.0427 (16)	0.0365 (14)	−0.0094 (13)	0.0078 (13)	−0.0080 (12)
C16	0.060 (2)	0.059 (2)	0.093 (3)	−0.0081 (18)	0.041 (2)	0.012 (2)
C17	0.0264 (12)	0.0272 (12)	0.0234 (11)	0.0000 (10)	0.0112 (10)	0.0046 (9)
N1	0.0286 (11)	0.0259 (10)	0.0239 (9)	0.0002 (8)	0.0134 (8)	−0.0030 (8)
N2	0.0269 (10)	0.0217 (9)	0.0255 (9)	0.0008 (8)	0.0162 (8)	−0.0003 (7)
N3	0.0375 (12)	0.0237 (10)	0.0282 (10)	0.0038 (9)	0.0174 (9)	0.0018 (8)
N4	0.0307 (11)	0.0285 (10)	0.0285 (10)	−0.0030 (9)	0.0105 (9)	−0.0001 (8)
Ni1	0.0277 (2)	0.0178 (2)	0.0207 (2)	0.000	0.01265 (18)	0.000
O1	0.0449 (13)	0.0956 (18)	0.0573 (14)	0.0003 (12)	0.0182 (11)	0.0297 (13)
S1	0.0482 (4)	0.0367 (4)	0.0449 (4)	0.0161 (3)	0.0250 (3)	0.0006 (3)

### Geometric parameters (Å, °)

**Table:**

C1—N1	1.326 (3)	C12—N4	1.359 (3)
C1—C2	1.395 (3)	C14—N4	1.449 (3)
C1—H1	0.9300	C14—H14A	0.9600
C2—C3	1.364 (4)	C14—H14B	0.9600
C2—H2	0.9300	C14—H14C	0.9600
C3—C4	1.399 (4)	C15—N4	1.458 (3)
C3—H3	0.9300	C15—H15A	0.9600
C4—C5	1.413 (3)	C15—H15B	0.9600
C4—C7	1.424 (4)	C15—H15C	0.9600
C5—N1	1.350 (3)	C16—O1	1.402 (4)
C5—C6	1.436 (3)	C16—H6	0.9600
C6—N2	1.368 (3)	C16—H9	0.9600
C6—C9	1.400 (3)	C16—H5	0.9600
C7—C8	1.346 (4)	C17—N3	1.146 (3)
C7—H7	0.9300	C17—S1	1.646 (2)
C8—C9	1.427 (3)	N1—Ni1	2.0569 (19)
C8—H8	0.9300	N2—Ni1	2.2556 (18)
C9—C10	1.406 (3)	N3—Ni1	2.047 (2)
C10—C11	1.353 (4)	Ni1—N3^i^	2.047 (2)
C10—H10	0.9300	Ni1—N1^i^	2.0569 (19)
C11—C12	1.422 (3)	Ni1—N2^i^	2.2556 (18)
C11—H11	0.9300	O1—H4	0.8217
C12—N2	1.346 (3)		
			
N1—C1—C2	122.5 (2)	H14B—C14—H14C	109.5
N1—C1—H1	118.8	N4—C15—H15A	109.5
C2—C1—H1	118.8	N4—C15—H15B	109.5
C3—C2—C1	119.3 (2)	H15A—C15—H15B	109.5
C3—C2—H2	120.3	N4—C15—H15C	109.5
C1—C2—H2	120.3	H15A—C15—H15C	109.5
C2—C3—C4	119.9 (2)	H15B—C15—H15C	109.5
C2—C3—H3	120.1	O1—C16—H6	109.5
C4—C3—H3	120.1	O1—C16—H9	109.5
C3—C4—C5	117.1 (2)	H6—C16—H9	109.5
C3—C4—C7	124.1 (2)	O1—C16—H5	109.5
C5—C4—C7	118.8 (2)	H6—C16—H5	109.5
N1—C5—C4	122.3 (2)	H9—C16—H5	109.5
N1—C5—C6	117.26 (19)	N3—C17—S1	179.6 (2)
C4—C5—C6	120.4 (2)	C1—N1—C5	118.7 (2)
N2—C6—C9	124.0 (2)	C1—N1—Ni1	125.71 (16)
N2—C6—C5	117.80 (19)	C5—N1—Ni1	115.34 (14)
C9—C6—C5	118.2 (2)	C12—N2—C6	117.53 (19)
C8—C7—C4	120.8 (2)	C12—N2—Ni1	131.28 (15)
C8—C7—H7	119.6	C6—N2—Ni1	107.06 (14)
C4—C7—H7	119.6	C17—N3—Ni1	171.3 (2)
C7—C8—C9	121.3 (2)	C12—N4—C14	120.6 (2)
C7—C8—H8	119.4	C12—N4—C15	119.6 (2)
C9—C8—H8	119.4	C14—N4—C15	113.4 (2)
C6—C9—C10	116.6 (2)	N3^i^—Ni1—N3	90.27 (11)
C6—C9—C8	120.1 (2)	N3^i^—Ni1—N1^i^	88.63 (7)
C10—C9—C8	123.3 (2)	N3—Ni1—N1^i^	93.08 (7)
C11—C10—C9	120.1 (2)	N3^i^—Ni1—N1	93.08 (7)
C11—C10—H10	119.9	N3—Ni1—N1	88.63 (7)
C9—C10—H10	119.9	N1^i^—Ni1—N1	177.57 (10)
C10—C11—C12	120.2 (2)	N3^i^—Ni1—N2	167.90 (7)
C10—C11—H11	119.9	N3—Ni1—N2	96.75 (7)
C12—C11—H11	119.9	N1^i^—Ni1—N2	100.76 (7)
N2—C12—N4	118.5 (2)	N1—Ni1—N2	77.31 (7)
N2—C12—C11	121.0 (2)	N3^i^—Ni1—N2^i^	96.75 (7)
N4—C12—C11	120.5 (2)	N3—Ni1—N2^i^	167.90 (7)
N4—C14—H14A	109.5	N1^i^—Ni1—N2^i^	77.31 (7)
N4—C14—H14B	109.5	N1—Ni1—N2^i^	100.76 (7)
H14A—C14—H14B	109.5	N2—Ni1—N2^i^	78.13 (9)
N4—C14—H14C	109.5	C16—O1—H4	106.5
H14A—C14—H14C	109.5		
			
N1—C1—C2—C3	−1.8 (4)	C6—C5—N1—Ni1	11.1 (2)
C1—C2—C3—C4	2.3 (4)	N4—C12—N2—C6	−173.0 (2)
C2—C3—C4—C5	1.1 (4)	C11—C12—N2—C6	6.8 (3)
C2—C3—C4—C7	−178.3 (2)	N4—C12—N2—Ni1	33.1 (3)
C3—C4—C5—N1	−5.3 (3)	C11—C12—N2—Ni1	−147.06 (18)
C7—C4—C5—N1	174.2 (2)	C9—C6—N2—C12	−0.8 (3)
C3—C4—C5—C6	175.4 (2)	C5—C6—N2—C12	179.39 (19)
C7—C4—C5—C6	−5.2 (3)	C9—C6—N2—Ni1	158.98 (18)
N1—C5—C6—N2	8.4 (3)	C5—C6—N2—Ni1	−20.8 (2)
C4—C5—C6—N2	−172.2 (2)	N2—C12—N4—C14	41.2 (3)
N1—C5—C6—C9	−171.4 (2)	C11—C12—N4—C14	−138.6 (2)
C4—C5—C6—C9	8.0 (3)	N2—C12—N4—C15	−168.5 (2)
C3—C4—C7—C8	179.1 (2)	C11—C12—N4—C15	11.7 (3)
C5—C4—C7—C8	−0.3 (4)	C1—N1—Ni1—N3^i^	−17.9 (2)
C4—C7—C8—C9	2.8 (4)	C5—N1—Ni1—N3^i^	155.82 (16)
N2—C6—C9—C10	−4.6 (3)	C1—N1—Ni1—N3	72.3 (2)
C5—C6—C9—C10	175.2 (2)	C5—N1—Ni1—N3	−113.98 (16)
N2—C6—C9—C8	174.7 (2)	C1—N1—Ni1—N2	169.6 (2)
C5—C6—C9—C8	−5.5 (3)	C5—N1—Ni1—N2	−16.74 (15)
C7—C8—C9—C6	0.1 (4)	C1—N1—Ni1—N2^i^	−115.3 (2)
C7—C8—C9—C10	179.4 (2)	C5—N1—Ni1—N2^i^	58.34 (17)
C6—C9—C10—C11	3.9 (4)	C12—N2—Ni1—N3^i^	137.6 (3)
C8—C9—C10—C11	−175.4 (2)	C6—N2—Ni1—N3^i^	−18.3 (4)
C9—C10—C11—C12	1.8 (4)	C12—N2—Ni1—N3	−97.3 (2)
C10—C11—C12—N2	−7.5 (4)	C6—N2—Ni1—N3	106.84 (14)
C10—C11—C12—N4	172.3 (2)	C12—N2—Ni1—N1^i^	−2.8 (2)
C2—C1—N1—C5	−2.3 (4)	C6—N2—Ni1—N1^i^	−158.71 (14)
C2—C1—N1—Ni1	171.22 (18)	C12—N2—Ni1—N1	175.7 (2)
C4—C5—N1—C1	5.9 (3)	C6—N2—Ni1—N1	19.77 (14)
C6—C5—N1—C1	−174.8 (2)	C12—N2—Ni1—N2^i^	71.63 (19)
C4—C5—N1—Ni1	−168.28 (17)	C6—N2—Ni1—N2^i^	−84.28 (14)

Symmetry codes: (i) −*x*+1, *y*, −*z*+1/2.

### Hydrogen-bond geometry (Å, °)

**Table:**

*D*—H···*A*	*D*—H	H···*A*	*D*···*A*	*D*—H···*A*
C14—H14C···S1^ii^	0.96	2.86	3.784 (3)	163
O1—H4···S1^iii^	0.82	2.65	3.331 (2)	142
C15—H15B···O1^iv^	0.96	2.51	3.427 (4)	161

Symmetry codes: (ii) −*x*+3/2, *y*+1/2, −*z*+1/2; (iii) *x*, *y*+1, *z*; (iv) −*x*+3/2, −*y*+1/2, −*z*+1.
